# Design and Characterization of a Three-Axis Hall Effect-Based Soft Skin Sensor

**DOI:** 10.3390/s16040491

**Published:** 2016-04-07

**Authors:** Tito Pradhono Tomo, Sophon Somlor, Alexander Schmitz, Lorenzo Jamone, Weijie Huang, Harris Kristanto, Shigeki Sugano

**Affiliations:** 1 Department of Modern Mechanical Engineering, School of Creative Science and Engineering, Waseda University, 3-4-1 Okubo, Shinjuku-ku, Tokyo 169-8555, Japan; sophon@sugano.mech.waseda.ac.jp (S.S.); schmitz@aoni.waseda.jp (A.S.); kou498431413@fuji.waseda.jp (W.H.); harris.kristanto@fuji.waseda.jp (H.K.); sugano@waseda.jp (S.S.); 2 Instituto de Sistemas e Robótica, Instituto Superior Técnico, Lisbon 1049-001, Portugal; ljamone@isr.tecnico.ulisboa.pt

**Keywords:** tactile, skin, sensor, magnetic

## Abstract

This paper presents an easy means to produce a 3-axis Hall effect–based skin sensor for robotic applications. It uses an off-the-shelf chip and is physically small and provides digital output. Furthermore, the sensor has a soft exterior for safe interactions with the environment; in particular it uses soft silicone with about an 8 mm thickness. Tests were performed to evaluate the drift due to temperature changes, and a compensation using the integral temperature sensor was implemented. Furthermore, the hysteresis and the crosstalk between the 3-axis measurements were evaluated. The sensor is able to detect minimal forces of about 1 gf. The sensor was calibrated and results with total forces up to 1450 gf in the normal and tangential directions of the sensor are presented. The test revealed that the sensor is able to measure the different components of the force vector.

## 1. Introduction

Tactile sensors are crucial for a safe and robust interaction of a robot with its environment. They provide the most direct measurements of the contact forces during planned and accidental contacts. In order to appropriately react to contact forces, it is beneficial if the force sensors are distributed in the robot skin and measure not only normal forces, but also shear forces. Moreover, soft skin is important for softening impact forces and also for robust object grasping. Several studies have been conducted in recent years to develop a distributed tactile sensor for robot skin [1]. For example, a soft capacitive-type sensor that could measure force in 3-axis was introduced in [2]. Although this sensor had high sensitivity and could measure normal and shear forces, each sensor required space of approximately 13 mm × 13 mm (from the center of one sensor to another). Moreover, the manufacturing required a lot of manual work.

In this paper we present a Hall effect–based tactile sensor that can measure three-directional force data in limited space. It is both physically small and easy to produce. We evaluate the feasibility of measuring 3-axis force while maintaining a soft exterior for safe interactions. While in previous work [3] we presented preliminary results obtained with the sensor, the current paper provides a detailed sensor characterization. In particular, drift due to temperature changes and the hysteresis in the sensor measurements are analyzed. Furthermore, a calibration to counteract crosstalk between the axes and to extract more precise force vector measurements is provided. Moreover, the design of a printed circuit board (PCB) is presented to show how 16 chips (each measuring 3-axis force) can be placed on a 20 × 23–mm-sized PCB.

The rest of this paper is organized as follows. In Section 2 we provide a review of related tactile sensors. Section 3 describes the sensor principle and the production process. Section 4 presents the experimental procedure that was used to evaluate the sensor and Section 5 shows the results. Section 6 shows a PCB for distributed sensing, and Section 7 draws conclusions and presents future work.

## 2. Related Works

There are many sensing principles which can be employed for tactile sensing [4]. This review will focus on sensors that are capable of measuring normal as well as shear forces in robotic skin. Touchence [5] sells a thin, small-sized 3-axis tactile sensor based on MEMS piezoelectric elements, but the sensor is rigid and the necessary additional electronics are bigger than the sensor itself. A small-sized optical sensor that can measure both normal and shear forces was already proposed more than 10 years ago in [6], but to the best of the authors’ knowledge it has not been integrated in a robotic system yet. Optical 3-axis sensors have also been integrated into soft sensor flesh [7], and those sensors have recently been made commercially available by Touchence as well. A smaller (10 mm wide and 8 mm high) but stiffer optical sensor that can measure the force vector is currently available from OptoForce [8]. An implementation of four tri-axial sensors for a robotic fingertip is presented in [9], and 3-axis F/T (force/torque) sensors were also integrated in the soft skin of the robot Macra [8], both based on strain-gauges. A capacitive 3-axis sensor embedded in soft silicone is presented in [2].

The idea of using Hall effect sensors and magnets to measure a tactile response was originally proposed in [10,11], where only preliminary prototypes were presented, and then this was not investigated anymore until recently [12,13,14,15,16]. In [14], Hall effect–based tactile sensors are integrated on a robot hand and used in object classification experiments; a full characterization of the sensor and more real world experiments are reported in [15]. However, the sensor is based on one-dimensional (1D) Hall effect sensing, and therefore only normal forces can be measured. The work in [12] instead proposes a three-dimensional (3D) sensor, with a design similar to the one we present; however, no accurate characterization is reported, and therefore it is not easy to evaluate the quality of the measurements. The work described in [13,16] is more mature, and it has been successfully applied to real robotic scenarios; however, even though many simulation analyses are presented, this work also lacks a complete characterization of the real sensor. Moreover, the design they propose (with a rubber dome and four Hall effect sensors) imposes constraints on the minimum size of the whole system. A magnetic-based tactile sensor for fingertips has been commercially produced [17]. However, the output signal from this sensor has to be amplified first before it can be read by a microcontroller. The amplifier has a rather big size, meaning that a lot of space is required for integrating this device into a robot. Instead, the current paper uses a single small-sized chip with digital output and the surface of the sensor is flat. Only a magnet embedded in soft silicone is required in addition in order to measure 3-axial forces.

## 3. Sensor Description

The sensor described in this paper is easy to produce and requires only a few tools. This section describes the sensor structure as well as the production process.

### 3.1. Sensor Concept

**Figure 1:**
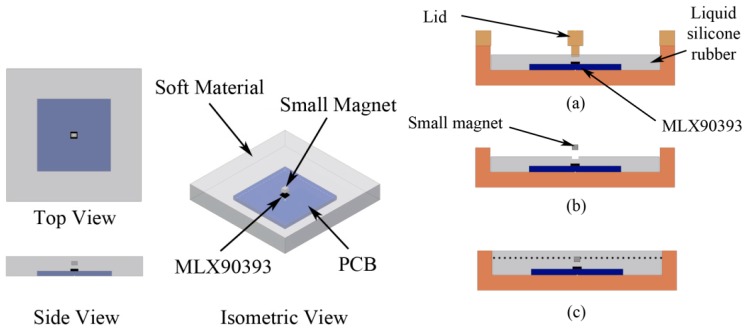
Conceptual design (left) and the molding process (right). (**a**) Liquid silicone rubber was poured into the molding cast; (**b**) A small magnet was placed inside the hole; (**c**) More liquid silicone rubber was poured above the first layer to cover the small magnet.

The force vector can be detected by measuring a magnetic field change. To achieve that, a magnetometer (MLX90393) from Melexis [18] is used. A single MLX90393 chip is capable of providing 3-axis magnetic data and temperature data through the I2C fast mode protocol (four wires). The chip is mounted on a printed circuit board (PCB). We embedded the chip below a soft material, specifically silicone rubber, and implanted a small magnet above it as shown in Figure 1. The soft material acts as a compliant layer, and also transmits the force applied on the top surface. As a result, the small magnet will be displaced from its initial position when force is applied, causing a magnetic field change. For the experiments in this paper, a PCB with one single chip is used. Section 6 of this paper shows how multiple chips can be implemented on one PCB.

### 3.2. Soft Outer Layer

**Figure 2:**
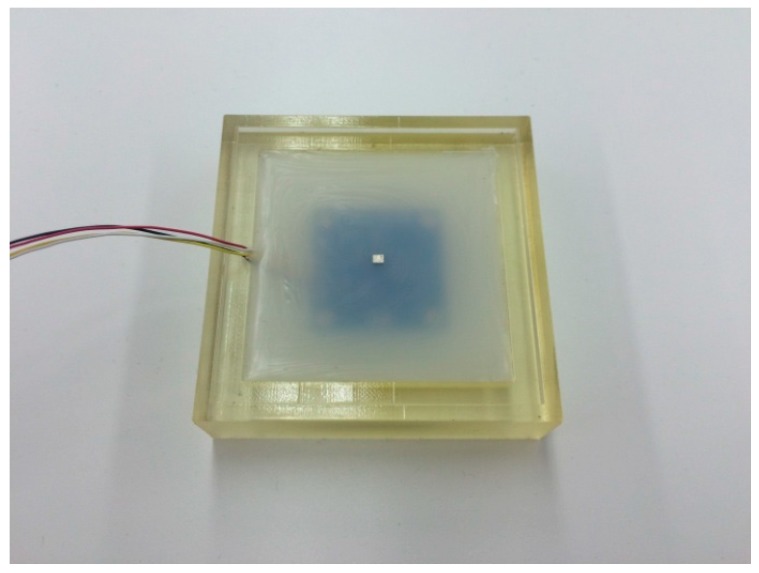
The prototype of the Hall effect–based skin sensor.

The following steps are required for the molding process. First, an MLX90393 chip is placed at the middle of a molding cast, supported with four guidance points and double-sided tape. The chip is covered by liquid silicone rubber (Ecoflex Supersoft from Smooth-On, shore hardness 00-30). Please note that optimal material selection and optimization of the thickness of the compliant layer is not the focus of this paper. To distribute the magnetic field evenly, the position of the magnet should be centered above the chip. A guidance lid (Figure 1a) is used to create a hole for placing a small magnet in the center. The magnet for the current implementation is a Neodymium magnet coated with nickel (Nd-Fe-B) with a dimension of 2 mm × 2.5 mm × 1.7 mm. After the first layer of silicone has cured, the magnet is placed inside the hole, and more liquid silicone rubber is used to cover it. The silicone layer above the PCB is 8 mm thick overall, with the small magnet covered by approximately 2 mm of silicone, as in Figure 2. In our experiments there was a good bond between the first and second layer of silicone. The silicone covers an area of 55 mm × 55 mm.

## 4. Experimental Setup

Three experiments were conducted to understand the characteristics of the sensor. This section explains the experimental setups and procedures that were used during the tests.

### 4.1. Temperature Drift Test

This test studies the effect of thermal drift on the sensor reading. The skin sensor was placed inside an oven along with a Sparkfun TMP102, which is an I2C temperature sensor for measuring the temperature inside the oven during the test. The MLX90393 chip also includes a temperature sensor. The experiment started with the room temperature of 27 °C, then the sensor was heated up until the skin sensor reached 40 °C. Afterwards, the oven was turned off and the door of the oven was opened to let the temperature drop to around 30 °C. The temperature value and the skin sensor’s readouts were recorded using Arduino Due, stored in an SD (secure digital) card.

### 4.2. Hysteresis Test

**Figure 3:**
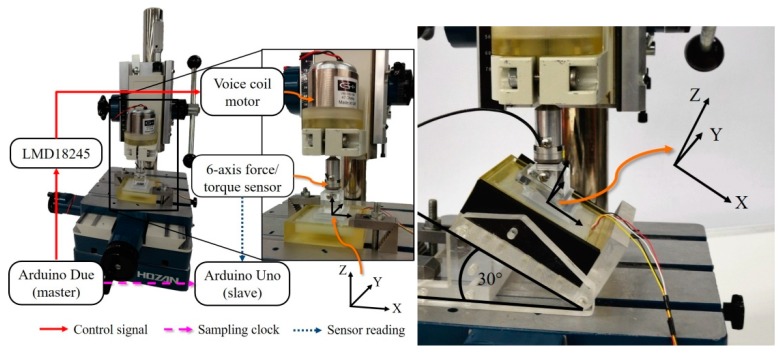
Experiment setup used in this paper. The right side shows the addition of the adjustable angle tilt stage and the angle push plate, in particular the 30-degree setup where the stage is adjusted to 30 degrees and the 30-degree push plate is used.

A viscoelastic material such as silicone can introduce hysteresis in the sensor’s force measurements. To evaluate the hysteresis, the skin sensor was placed on top of an acrylic platform table tilted 45 degrees in the y-axis direction. The sensor was pushed for 5 min with a load of 1450 gf (the maximum load that our experimental setup can achieve). To perform this, a voice coil motor (VM5050-190 from Geeplus), a linear bushing, an aluminum shaft adapter, a six-axis force/torque (F/T) sensor (Nano1.5/1.5 from BL Autotech) for monitoring the pushing force during the experiment, and a 30 × 30 mm acrylic push plate, which is used to push on the proposed sensor, were utilized. The configuration for this test can be seen in Figure 3. Two microcontrollers were required due to the different input voltage of our sensor (3.3 V) and the F/T sensor (5 V). The data from both sensors were recorded into the SD cards installed on Arduino Due and Uno with the synchronized sampling rate of 100 Hz in our experiments (the maximum sampling rate of the Hall effect sensor is about 240 Hz to measure all three axes). Finally, the voice coil motor applied no force on the sensor for one minute, and afterwards the acrylic push plate was retracted. The silicone is sticky, and therefore a force in the minus z direction is recorded when the acrylic push plate is removed.

### 4.3. Load Test

To calibrate the sensor and to evaluate its capability of tri-axial force measurement, two experiments were conducted. The first experiment was a normal force test where multiple magnitudes of normal force were applied on the sensor’s top surface. In the second experiment, the sensor was pushed with both normal and shear force in different angles and with different force magnitudes.

The configuration for this experiment was similar to the hysteresis test. In the normal force experiment, the skin sensor was mounted directly on the flat and sturdy X-Y table. In the shear force experiment, the sensor was mounted on an adjustable angle tilt stage (Figure 3). This acrylic stage was fixed to the X-Y table. The angle can be adjusted in three different positions that are 15, 30, and 45 degrees, and corresponding acrylic push plates with the same angles were used.

In both the normal and shear force experiments, the sensor was pushed by the voice coil motor with a stepwise force and its magnitude was increased every 10 seconds. The applied force was ranging from approximately 70 gf to 1450 gf. A Savitzky-Golay filter was utilized to filter all data (polynomial order = 4, frame size = 21). We performed the normal force test and the shear force tests with 15, 30, and 45 degree, in four directions (+/− x/y direction).

## 5. Results and Discussion

### 5.1. Thermal Drift Evaluation

**Figure 4:**
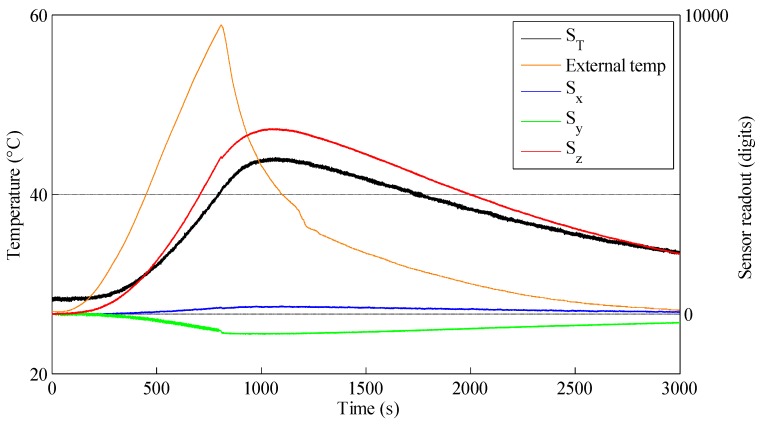
The effect of temperature changes on the sensor measurement.

From Figure 4 it can be clearly seen that the Hall effect sensor measurements change with the changing temperature, even though the temperature change measured by the chip was slower than the external temperature. A possible explanation for the change in the Hall effect measurements is an expansion of the silicone packaging with higher temperature. The test also revealed that the sensor reading in the z-axis was the most affected by the temperature change, but also the x-axis and y-axis measurements slightly changed; a closer look reveals that the y-axis is more affected than the x-axis. This is in accordance with the results presented in Section 5.3, which show that changes in the z-axis also affect the x-axis and y-axis, which might be due to a slightly misaligned magnet. The graph shows that the sensor changes are proportional to the temperature change measured by the chip, meaning that temperature compensation can possibly be performed.

A linear regression was conducted to find the coefficient k for calibrating the sensor’s outputs. We selected a Huber robust model for this task. The temperature compensation was calculated as follows:

(1) $$S_{i,T}{}_{}=S_{i}-k_{i}\times \Delta S_{T}$$

where:

• *i* is each axis of the skin sensor (x, y and z).
• *S_i_* and *S_i,T_* are the skin sensor readout and compensated value, respectively.
• Δ*S_T_* is the temperature change measured by the MLX90393 built-in temperature sensor.

**Figure 5:**
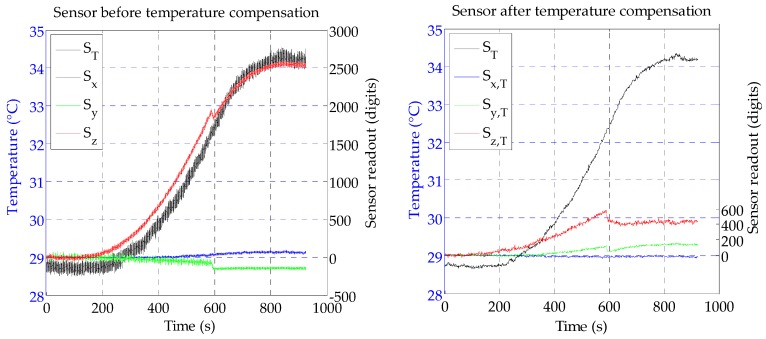
A comparison between the sensor’s readout before and after temperature compensation was applied. The right figure also shows the moving average of the sensor temperature, which was used for calibrating the sensor.

To evaluate the temperature compensation performance, another test was conducted. This time, the temperature was raised to 35 °C. Figure 5 shows a comparison between the sensor’s readout before and after the compensation was applied. A moving average of the temperature was used for the compensation. After being compensated, the z-axis maximum value was around 600 digits (over a full scale of 65,500 digits), which corresponds to 120 g in our experiments (contact size 30 × 30 mm). Further improvements are likely possible by employing a high-pass filter. Furthermore, small steps can be seen on S_y_ and S_z_. The cause of those is not clear to the authors and will be further investigated in future work.

### 5.2. Hysteresis Evaluation

**Figure 6:**
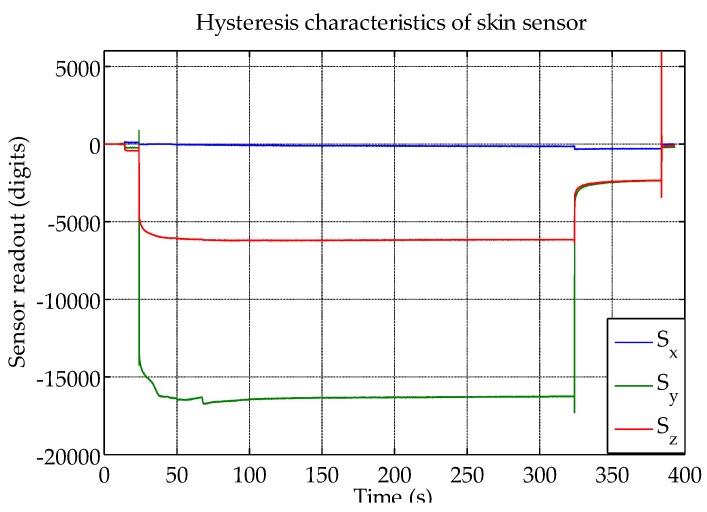
Hysteresis characteristics of the skin sensor.

As expected, there is hysteresis in the sensor measurements. In the result presented in Figure 6, it took about one minute for the sensor to reach its quasi-static state, both while loading and unloading the sensor. The x-axis, which was not loaded, showed only a minor drift. Please note that an optimal soft material selection was not the focus of this study, and the hysteresis can probably be reduced with different materials, for example closed-cell foams.

### 5.3. Load Tests and Calibration

**Figure 7:**
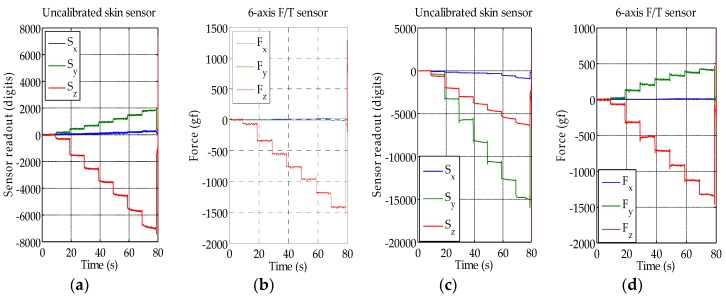
The sensor’s readout (**a**) and the corresponding force from F/T sensor (**b**) when only normal force is applied; The sensor’s readout (**c**) and the corresponding force from F/T sensor (**d**) when 45-degree shear force is applied in the y-direction.

The results from the load test can be seen in Figure 7. Figure 7a,b show the Hall effect and reference force sensor readout, respectively, when only a normal force is applied. Even though only normal force was applied, the Hall effect sensor also detected a magnetic field change in the y-axis. A related effect is also described in Section 5.1, and we suspect that the orientation of the small magnet was not perfectly aligned with the sensor and caused this. Furthermore, as silicone is soft but incompressible, forces in one axis can cause movements in another direction, as the silicone moves away from the pressure.

Figure 7c shows the Hall effect sensor response when a 45-degree shear force is applied in the y-axis. Even though no force was applied in the x-axis, as can be also seen from the measurements of the reference sensor in Figure 7d, the Hall effect sensor also detected a small magnetic field change in the x-axis direction. Furthermore, the y-axis Hall effect sensor measurements are bigger than the z-axis measurements, even though the force in the z-axis was bigger. Due to this cross-talk and different magnitude of the skin sensor response, a calibration was performed.

**Figure 8:**
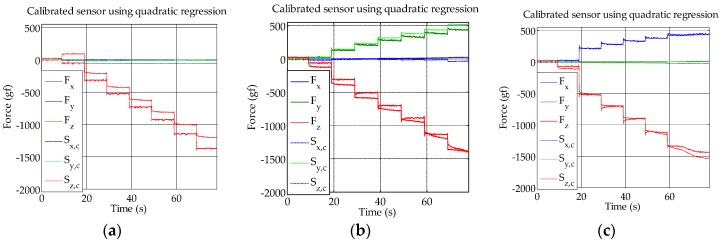
(**a**) Normal force; (**b**) 45-degree (y-axis direction); (**c**) 45-degree (x-axis direction) force calibration result. S_x,c_, S_y,c_ and S_z,c_ are the calibrated skin sensor measurements.

We used different models to calculate the x, y and z forces from the Hall effect sensor values, with the measurements of the six-axis F/T sensor as the reference. To calculate the parameters for the calibration, datasets from all angles were used. For the evaluation purpose, we used new datasets that were not used for the calibration. Robust Huber regression was used (MATLAB function LinearModel.fit); least squares regression performed nearly the same as the robust Huber regression. A quadratic model performed better than a linear model, as can be seen by higher R-square values in Table 1. Also, a neural network (one hidden layer with 20 hidden units) was trained with the same training data that we used for approximating the parameters of the linear or quadratic equation. The neural network performed better for the test case with only normal force, but overall the quadratic equation performed best. The calibration result using the quadratic model is shown in Figure 8. The graphs show the comparison between the force detected using the F/T sensor and the force calculated using the skin sensor. A good correspondence between the measurements can be observed, with the biggest differences in the normal load case for the z-axis.

**Figure 9:**
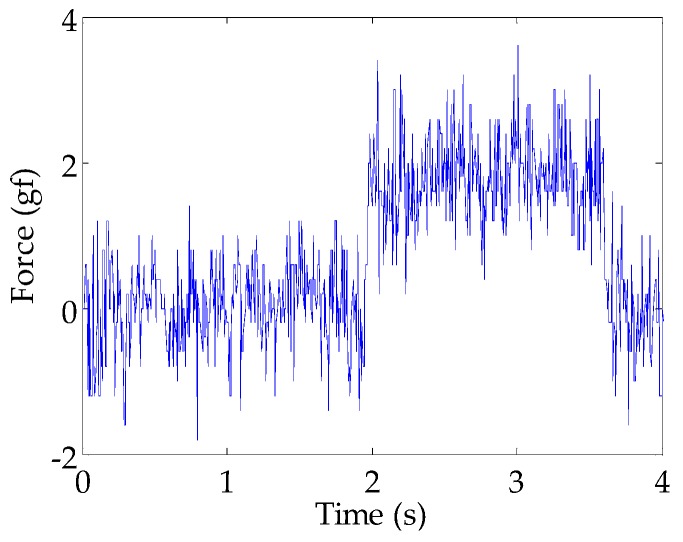
The z-axis calibrated sensor measurements (S_z,c_) when a weight of 1 g (contact area of about 1 cm^2^) is placed on the sensor at a time of around 2 s. The calibration is slightly incorrect in this case, showing a measurement of 2 gf. This might be partially due to the different contact area than that during the calibration. Nevertheless, it can be seen that the sensor can already detect a 1 g weight.

As a final evaluation, the minimum detectable load was evaluated. Rubber weights with a diameter of about 1 cm were placed on the sensor and it was found that a force of about 1 gf in the z-axis produced sensor measurements that are higher than the observed noise of the sensor, as can be seen in Figure 9.

## 6. Design of PCB for Distributed Sensors

**Figure 10:**
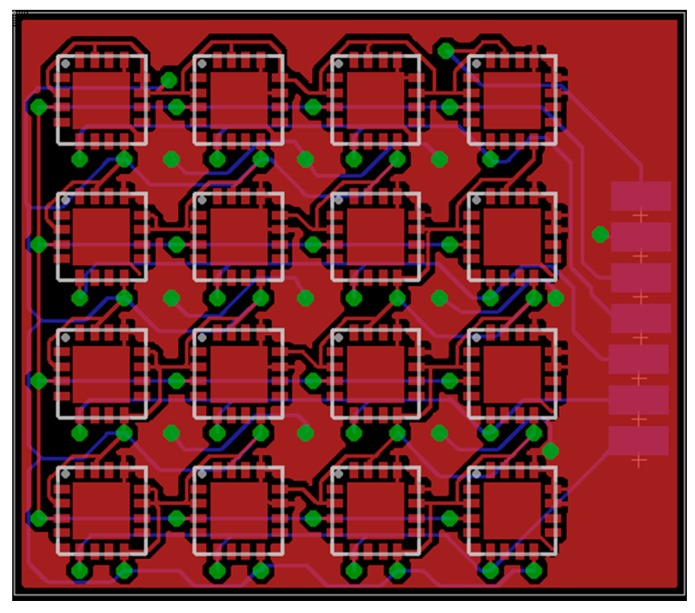
A custom PCB for integrating 16 MLX90393 chips.

A custom PCB (Figure 10) has been designed to integrate 16 MLX90393 chips for detecting distributed force vectors. The PCB has two layers, is only 20 × 23 mm big and has four I2C outputs. The distance of the center of one chip to the next one is 4.7 mm. After the PCB has been produced, in future work the crosstalk between the sensors will be evaluated.

## 7. Conclusions

This paper presented the design and characterization of a Hall effect–based soft skin sensor. The temperature test shows that the skin sensor’s readout in the z-axis direction was the one mostly affected by temperature changes. Using a built-in temperature sensor, drift compensation was performed. Next, the hysteresis was evaluated. Indeed there is hysteresis in the sensor measurements, which is to be expected due to the use of silicone, which is a viscoelastic material. After the calibration of the sensor, when applying varying amounts of normal and shear force, the tests showed that the sensor can measure the components of the force vector. Furthermore, the design of a PCB with 16 3-axis force measurements in limited space is presented. As future work, the crosstalk between those 16 sensors in close proximity will be evaluated. Furthermore, a spherical magnet will be used in order to avoid possible spurious signals on irrelevant axes. The use of different materials or different thicknesses of the packaging will be studied as potential means to reduce the hysteresis.

## Figures and Tables

**Table 1:** R-squared value for the normal force and shear force experiments.

	Linear + Huber	Quadratic + Huber	Feedforward Neural Network
Normal Force	0.8634	0.8925	0.9368
Shear 45—y	0.8634	0.9418	0.8275
Shear 45—x	0.9272	0.9744	0.9644

## Abbreviations

The following abbreviations are used in this manuscript:

PCB	Printed circuit board
MEMS	Microelectromechanical system
F/T	Force/torque
SD	Secure digital
